# Development of A New Delivery System Based on Drug-Loadable Electrospun Nanofibers for Psoriasis Treatment

**DOI:** 10.3390/pharmaceutics11010014

**Published:** 2019-01-04

**Authors:** Leticia Martínez-Ortega, Amalia Mira, Asia Fernandez-Carvajal, C. Reyes Mateo, Ricardo Mallavia, Alberto Falco

**Affiliations:** Institute of Research, Development and Innovation in Biotechnology of Elche (IDiBE) and Molecular and Cellular Biology Institute (IBMC), Miguel Hernández University (UMH), 03202 Elche, Spain; leticia.martinez@goumh.umh.es (L.M.-O.); a.mira@umh.es (A.M.); asia.fernandez@umh.es (A.F.-C.); rmateo@umh.es (C.R.M.)

**Keywords:** PMVE/MA, electrospinning, nanofibers, capsaicin, psoriasis, TRPV1

## Abstract

Psoriasis is a chronic autoimmune systemic disease with an approximate incidence of 2% worldwide; it is commonly characterized by squamous lesions on the skin that present the typical pain, stinging, and bleeding associated with an inflammatory response. In this work, poly(methyl vinyl ether-*alt*-maleic ethyl monoester) (PMVEMA-ES) nanofibers have been designed as a delivery vehicle for three therapeutic agents with palliative properties for the symptoms of this disease (salicylic acid, methyl salicylate, and capsaicin). For such a task, the production of these nanofibers by means of the electrospinning technique has been optimized. Their morphology and size have been characterized by optical microscopy and scanning electron microscopy (SEM). By selecting the optimal conditions to achieve the smallest and most uniform nanofibers, approximate diameters of up to 800–900 nm were obtained. It was also determined that the therapeutic agents that were used were encapsulated with high efficiency. The analysis of their stability over time by GC-MS showed no significant losses of the encapsulated compounds 15 days after their preparation, except in the case of methyl salicylate. Likewise, it was demonstrated that the therapeutic compounds that were encapsulated conserved, and even improved, their capacity to activate the transient receptor potential cation channel 1 (TRPV1) channel, which has been associated with the formation of psoriatic lesions.

## 1. Introduction

Psoriasis is a chronic immune-mediated disease that affects 2% of the population, comprising different clinical manifestations, which are mainly characterized by skin disorders such as erythematous papules or scaly teardrop-like lesions associated with the characteristic pain, stinging, and even bleeding of an inflammatory process. The most affected areas are usually the elbows, knees, scalp, and lower back [1,2].

The immune system plays a crucial role in the pathogenesis of the disease, and particularly, the deregulation of the crosstalk between the innate and adaptive immune system within the interleukin-23 (IL23)/T helper cell 17 (Th17) axis [1,3,4]. Thus, the therapeutic treatments focus on targeting the Th17 response, either as local or systemic non-specific immunosuppressors (conventional therapies) such as dermocorticoids and methotrexate. The side effects produced by these treatments have boosted the use of antibody-based systemic treatments that recognize, bind, and block the activity of those cytokines involved in the Th17 response (biological therapies), such as IL17A (Ixekizumab, Taltz™; Secukinumab, Cosentyx™), IL23 (Guselkumab, Tremfya™), IL23 and IL12 (Ustekinumab, Stelara™), and TNF-α (Adalimumab, Humira™; Etanercept, Enbrel™; Infliximab, Remicade™) [1,5,6]. However, despite the promising results offered by this type of treatment, they also have limitations such as their high cost, some adverse effects, a loss of efficacy due to the development of anti-drug antibodies, and the inability to avoid occasional symptomatic outbreaks or to act on some poorly accessible locations [1,7,8,9]. Therefore, there is still a need to use therapeutic agents to alleviate the discomfort derived from the symptoms caused by the psoriatic lesions [1,6,10].

The main palliative agents for the dermal symptomatology of the disease are keratolytics, to reduce the generation of scales, and analgesics, to relieve the painful and itching sensation. Among the keratolytics, salicylic acid stands out, which acts by lowering the pH of the stratum corneum and thus preventing the adhesion of keratinocytes [11]. In turn, it also improves the absorption of other topical treatments by reducing the stiffness of the stratum, and also reduces the pruritus [12].

In terms of analgesic therapy, one of the most common targets in topical applications is the transient receptor potential cation channel 1 (TRPV1), commonly called the capsaicin receptor. This receptor is mainly found in the nociceptive neurons of the peripheral nervous system, although they have also been described in other non-neuronal tissues, including skin cells. TRPV1 is a non-selective tetrameric cation channel with high permeability to calcium (Ca^2+^) that has been involved in the transmission and modulation of pain when triggered by different physical and chemical stimuli such as temperatures above 43 °C, acidic pH, capsaicin, and some endogenous mediators of inflammation [13,14,15]. Capsaicin, an alkaloid present in several species in the genus *Capsicum*, and some of its derivatives act as analgesic agents by producing TRPV1 desensitization [13,16]. Additionally, this family of compounds hold other biological properties such as cardiovascular stimulator, antioxidant, and anticancer, as well as anti-inflammatory, by for instance suppressing the inducement of TNF-α, which is also a psoriasis mediator [17,18,19,20]. Methyl salicylate, which is derived from salicylic acid, is another analgesic agent. This compound belongs to the family of non-steroidal anti-inflammatory drugs (NSAIDs), and has also been reported to activate TRPV1 [21,22]. In addition, it is easily absorbed through the skin, and is already used for the treatment of inflammation and pain [22].

However, for the maximization of the expected benefits of their combined delivery, it would be necessary to have a suitable system for their topical application, and desirably, an encapsulation procedure that allows combining all three compounds together without losing their activity, in addition to being simple and scalable to industrial production. In this sense, recent advances in nanotechnology applied to the field of medicine offer an alternative that meets all these requirements, i.e., polymer nanofibers produced by the electrospinning technique [23,24,25]. In addition, the correct choice of the integrating materials would allow the development of nanostructures with appropriate release properties according the particular dosage needs of their load, which in the specific case of capsaicin should be sustained in order to keep TRPV1 desensitized for a prolonged period.

In this work, based on the progress made in our previous study [26], the production process of electrospun nanofibers loaded with all three compounds described above is optimized using poly(methyl vinyl ether-*alt*-maleic ethyl monoester) (PMVEMA-ES) as the polymeric material. Such a biodegradable, biocompatible, bioadhesive, and low-toxic polymer is already commercialized, and used in other biomedical applications, and has already shown its suitability for the creation of these nanostructured systems with the appropriate release characteristics [26,27,28,29,30]. Additionally, this polymer not only allows the possibility of being used as the main building polymer for the creation of nanofibers, but also allows combining with other materials, such as fluorene-based copolymers [31,32], in order to combine functional properties within the same nanostructure [26,33,34]. The morphological characterization of these nanofibers, together with their encapsulation capacity, stability over time, and activity of the encapsulated compounds are also analyzed in this study.

## 2. Materials and Methods

### 2.1. Polymers, Solvents, Drugs, and Common Materials

The copolymer of poly(methyl vinyl ether-*alt*-maleic acid) monoethyl ester (PMVEMA-ES) (*M*_w_: ~130 kg/mol) was supplied in a 50% *w*/*w* solution in ethanol (Sigma-Aldrich, Saint Louis, MO, USA).

The solvents used were dichloromethane, acetone, ethanol (Merck KGaA, Darmstadt, Germany), methanol (VWR International, Radnor, PA, USA), sulfuric acid, and anhydrous dimethyl sulfoxide (DMSO) (Sigma-Aldrich).

The reagents used were salicylic acid, capsaicin, ruthenium red (RR) (Sigma-Aldrich), magnesium sulfate anhydrous (Honeywell Fluka, Morris Plains, NJ, USA), sodium hydroxide (Panreac AppliChem—ITW Reagents, Cinisello Balsamo, Milan, Italy), and ferric chloride (Sigma-Aldrich). Although it is considered in the text as a unique therapeutic agent, according to the supplier’s analysis certificate, the purity of the commercial capsaicin used in this work was 61.1%, and it contained 31.2% of dihydrocapsaicin, which is a derivative with very similar chemical and biological properties to capsaicin.

Methyl salicylate was synthesized from the esterification of salicylic acid in a solution of methanol and sulfuric acid by following the method proposed by Carrillo-Arcos *et al.* (2016) [35]. Briefly, the 2-hydroxybenzoic acid (28.96 mmol) was dissolved in 60 mL of methanol, and then sulfuric acid (98%, 2 mL) was added dropwise to the solution, and the mixture was stirred at reflux for 18 h. Next, the solvent of the reaction was removed by vacuum rotary-evaporation, and the crude product was neutralized to pH 5–6 with a solution of NaOH (1 M, 50 mL), which was extracted three times with dichloromethane (3 × 50 mL). The combined organic phases were dried (magnesium sulfate) and concentrated (rotavap). The crude oil was distilled in a Kugelrohr B-585 glass furnace (Büchi, Flawil, Switzerland) to finally obtain a colorless oil (yield of 75%).

### 2.2. Electrospinning

The preparation of nanofibers was performed by means of the methodology described before by Mira *et al.* (2017) [26]. After optimization, an electrospinnable solution containing 25% *w*/*w* of PMVE/MA-ES in ethanol was selected. As for the active agents, a combination of 1.5% salicylic acid, 1% capsaicin (2:1 together with dihydrocapsaicin), and 1% methyl salicylate, all *w*/*w* with respect to the polymer weight, was used. 

The electrospinning process was performed on a device that included a two-mL Discardit II syringe (Becton Dickinson, Franklin Lakes, NJ, USA) through which the polymer solution was introduced, and from which it was pumped through a blunt-end stainless steel hypodermic needle 316 of 20 Gauge (101.6 mm in length, external diameter 0.902 mm, and internal diameter 0.584 mm) (Sigma-Aldrich) at a sustained flow controlled by a KDS 100 infusion pump (KD Scientific, Holliston, MA, USA). The needle and the aluminum foil collector, faced vertically, are connected to a Series FC high voltage source (Glassman High Voltage Inc., Whitehouse Station, NJ, USA), which provides the voltage responsible for the generation of the jet to be deposited on the collector located at a settled distance. Any material that is intended to be covered with a mat of electrospun nanofibers, as for example in this work, the adhesive dressings, would be placed on the aluminum collector. After the optimization of the operational parameters (see corresponding section for further details), values were selected for the elaboration of the batches of nanofibers for the following experiments. 

### 2.3. Microscopy

For the observation in optical microscopy, the nanofibers were electropun on a microscope slide (Deltalab, Barcelona, Spain) arranged on top of the aluminum collector. The inverted fluorescence optical microscope that was used was a Microsystems DMI3000B (Leica, Bensheim, Germany) equipped with a Leica EL6000 compact light source and a Leica DFC 3000G digital camera. The images were taken with a 63× objective in phase contrast, and the image processing was done manually using the program Leica Application Suite AF 6000 Module Systems. By this methodology, an initial screening, and then a more exhaustive screening, were carried out. 

After the optimization of the electrospinning parameters, selected nanofiber samples were also analyzed by scanning electron microscopy (SEM), without metal coating, in a JSM-6360 LV device (Jeol, Tokyo, Japan). In both cases, from the images obtained for each sample, a total of 100 measurements of nanofiber diameters were taken and analyzed by using the Image J software (National Institutes of Health, NIH, Bethesda, MD, USA).

### 2.4. Gas Chromatography and Mass Spectrometry (GC-MS)

The amount of the therapeutic agents contained in the nanofibers, which are necessary for analyzing the encapsulation efficiency and the stability of the encapsulation over time, was determined by means of a gas chromatograph coupled to a mass spectrometer, in particular, a GCMS-QP2010 SE equipment with quadrupole detector supplemented with a thermal TD-20 adsorption attachment and an AOC-20i/s automatic sample injector (Shimadzu, Kyoto, Japan). The employed GC column was an Agilent J&W capillary HP-5MS UI (5% diphenyl–95% dimethylpolysiloxane, 30 m × 0.25 mm id, film thickness 0.25 μm) (Agilent Technologies Inc., Santa Clara, CA, USA).

The procedure of this analytical methodology is based on a previous study [36]. Briefly, the temperatures of the injector and detector were 250 °C and 210 °C, respectively. Helium was used as a carrier gas at a flow rate of 1.5 mL/min. The chosen program was 40 °C for two minutes; then, the temperature was raised at a speed of 10 °C/min to 240 °C and, thereafter, at a rate of 5 °C/min up to 270 °C, which was finally maintained for five minutes.

In each experiment, a calibration curve was created for each of the analyzed compounds whose concentration ranges were between 0.018–1.420 mg/mL for capsaicin, 0.009–0.720 mg/mL for dihydrocapsaicin, 0.04–3.22 mg/mL for methyl salicylate, and 0.161–3.22 mg/mL for salicylic acid. All of the values that were provided for each curve corresponded to the area under the curve that was obtained for each concentration of the corresponding compound. In this way, the curves were preferably adjusted to a quadratic equation correlating area with concentration *R*^2^ coefficients greater than 0.99 in all of the cases (see Figure S1 in supplementary information).

The analyzed samples corresponded to two independent productions of nanofibers loaded with the three therapeutic agents as described above. Each sample of nanofibers was analyzed when just prepared and at 5 and 15 days post-production. These samples were stored in a drawer, uncovered, protected from light, and at room temperature. Immediately prior to their analysis, the samples were dissolved in dichloromethane, filtered with 0.2-μm nylon membranes (Millipore, Bedford, OH, USA) and injected at a concentration of 10 mg/mL *w*/*v*. As controls, nanofibers without encapsulated compounds and fresh polymer solutions (made just prior to the electrospinning process) were used. 

### 2.5. Cell Culture

For *in vitro* cell assays, human embryonic kidney cells stably expressing TRPV1 (HEK293-VR1) were used. The cells were maintained in Dulbecco’s modified Eagle’s medium (DMEM) supplemented with 10% *v*/*v* fetal bovine serum (FBS) (Sigma-Aldrich) and 50 μg/mL gentamicin (Thermo Fisher Scientific, Waltham, MA, USA). Cells were cultured in 25-cm^2^ flasks at 37 °C in a humidified atmosphere with 5% CO_2_, and for their harvest they were detached with 0.25% trypsin-EDTA solution. For the assays, cells were seeded in opaque 96-well plates with transparent bottoms (Corning Incorporated, Corning, NY, USA) at a cell density of 40,000 cells three days before treatment.

### 2.6. TRPV1 Channel Activity Assays

Since the TRPV1 channel allows the non-selective passage of cations such as Ca^2+^ when activated, a Ca^2+^ fluorescence probe has been used to indirectly quantify such activation, which might be induced by the experimental treatments. Thus, Ca^2+^ fluorography assays were performed by using the probe Fluo-4 NW (Molecular Probes-Thermo Fisher Scientific, Waltham, MA, USA) supplemented with 2.5 mM of probenecid, which improves the permeation of the probe in the cells and inhibits losses of fluorescence by avoiding its exit to the cellular exterior, as was recently reported by Serafini *et al.* (2018) [37].

Briefly, in cell plates prepared as described in the previous section, the culture medium was removed, and 100 μL/well of probe solution was added and incubated for 30 min at 37 °C followed by 30 min at 30 °C, always in the dark. Then, the fluorescence measurements were taken (excitation at 485 nm and emission at 535 nm) with a POLARstar Omega plate reader (BMG LABTECH GmbH, Offenburg, Germany) for 20 cycles (each one corresponding to a period of 156 s) and at a constant internal temperature of 30 °C. After the third cycle, one μL/well of each formulation that had been previously dissolved in DMSO at the corresponding concentration was added manually in order to reach the expected treatment doses in the well. In this sense, nanofibers were dissolved at 50 mg/mL in order to treat cells with 54 μM, 33 μM, and 10 μM of salicylic acid, methyl salicylate, and capsaicin, respectively. A solvent control (DMSO), capsaicin as agonist compound at 10 μM [38], and RR as a non-competitive inhibitor [39] at 10 μM were also included. Then, the measurements continued until cycle 20. After cycle 10, one μL of 10 μM capsaicin in DMSO was added to some wells that had been previously treated with RR in order to check the stability and selectivity of the system. Calibration curves for capsaicin and methyl salicylate were also included (range 0.1–30 μM and 15–120 μM, respectively). Each sample was analyzed in triplicate in each assay. 

### 2.7. Preliminary In Vivo Test of Topical Application

An initial *in vivo* study was carried out with three healthy human volunteers to evaluate, when administered topically, the stability time of the experimental nanofibers, as well as to determine the functionality of the encapsulated TRPV1 agonists by analyzing the responsiveness to them. Each volunteer was given an adhesive dressing with electrospun nanofibers on each arm on the skin surface of the deltoid. This test was blind, since for each volunteer, one of the dressings contained empty nanofibers, and the other contained drug-loaded ones, but they were assigned randomly to each of their arms. Each dressing contained approximately 25 mg of nanofibers distributed over a surface area of 20 cm^2^. The duration of the treatment was eight hours. The surface of the dressing was kept protected from light throughout the full period. Every two hours, photographs were taken of each dressing to determine the state of the nanofibers and treated skin. At the end of the trial, each volunteer was asked which dressing they thought contained the TRPV1 agonists. This study was approved by the Project Evaluation Board of Miguel Hernández University (approval no. 2018.12.05.FPRL).

### 2.8. Data Analysis and Graphics

Data were analyzed by either GraphPad Prism v6 and Microsoft Excel software. Both of them were used for creating the graphs. Statistical analysis was performed with GraphPad Prism v6 specifically. Applied statistical methods are stated together with the analyzed data in the Results section.

## 3. Results and Discussion

### 3.1. Optimization of PMVE/MA-ES Nanofibers Encapsulating Three Therapeutic Agents

Initially, we proceeded to the optimization of the morphology and size of the nanofibers without the encapsulated agent. For this, the operational values of the electrospinning procedure were modulated within a range that had been estimated from our previous study [26]. They included a concentration of PMVEMA-ES (20–28% *w*/*w*), pumping flow (0.25–1.35 mL/h), voltage (6–17.5 kV), and distance between the needle and collector (8–30 cm). 

**Figure 1:**
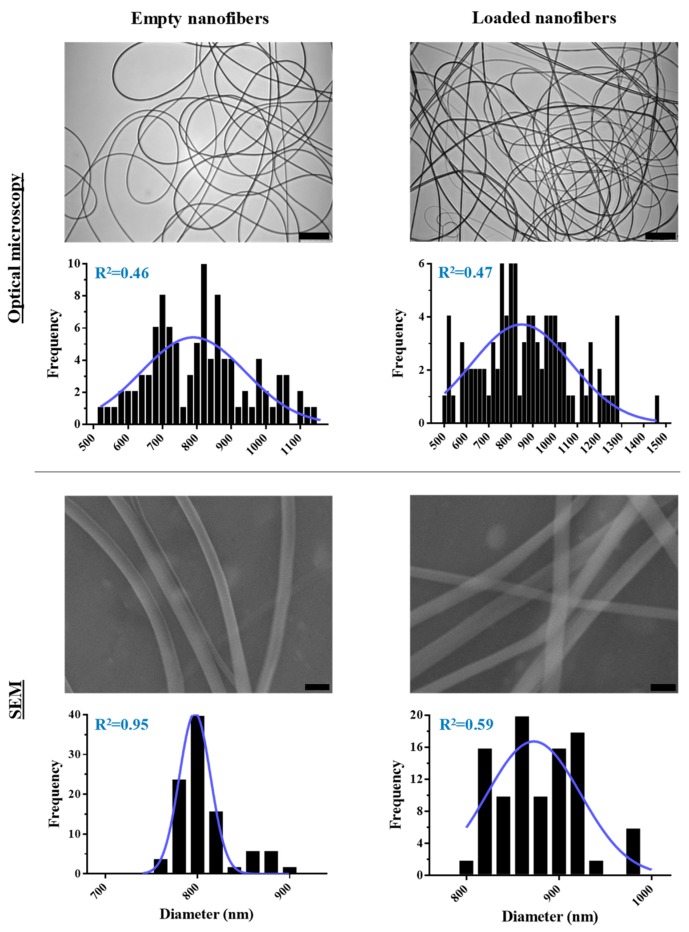
Optical microscopy and SEM analysis of optimized nanofibers. Representative optical microscopy and SEM images that include either empty or loaded nanofibers obtained by using optimized electrospinning parameters are shown (scale bars: 25 μm for optical microscopy, and 1 μm for SEM images). Corresponding frequency histograms of their diameters are also included. Each histogram was performed from the data obtained from several images until reaching 100 measurements. Best-fit adjustments (and their R^2^) to a Gaussian distribution are indicated in blue.

The concentration of polymer was shown to be determinant for the morphology and size of the final nanofibers. Independently of the other parameters, the values of greater uniformity and smaller fiber diameter were obtained with a concentration of 25% *w*/*w* of PMVE/MA-ES (as in our previous study [26]). Therefore, the concentration of polymer was fixed at this value for the analysis of the rest of the operational parameters, which were shown to be inversely proportional to the size of the nanofibers until reaching a critical value at which abrupt increases in size or the appearance of morphological aberrations could be observed. In short, the combination of values with which the smallest uniform fibers (810 ± 141 nm) were obtained were 15.5 kV, 12 cm, and 0.25 mL/h (Figure 1). However, when adding the therapeutic agents, nanofibers were significantly larger (about 200 nm more) by using these same parameters; the reason we proceeded to refine them again under these new circumstances was in order to reduce the size of the nanofibers below the micron. Thus, the optimal values to obtain uniform nanofibers of 878 ± 209 nm were 17 kV voltage, an eight cm needle–collector distance, and a flow of 0.25 mL/h (Figure 1). 

So far, the analysis of the nanofibers had been done by optical microscopy; however, to analyze the morphology in more detail and with greater precision, the size of the optimized nanofibers was observed by SEM. The measurements obtained by electron microscopy are equivalent to those obtained by optical microscopy: 808 ± 31 nm for empty nanofibers, and 875 ± 49 nm for those with encapsulated therapeutic content (Figure 1). 

The low amount of encapsulated drug that was used in this work (3.5% all together) does not explain the increase in size that was observed in the loaded nanofibers, even more considering that loading percentages are usually much higher for molecules that have a similar complexity, which even reduces nanofiber diameters [40,41]. Neither did an incompatibility in solubility, since all three compounds are soluble in the polymer-solvent system that was used. In fact, this aspect has been reported as critical, as well as the adequate selection of the solvent [42,43], for the morphology of fibers [44]. In this sense, from a broad perspective, the factors influencing fiber morphology are classified into two main groups: the processing and solution ones (briefly reviewed in [45]). Thus, while the processing factors are associated with the electrospinning mechanical set-up and environmental conditions, such as relative humidity, the solution factors refer to the physicochemical characteristics of the electrospinning solution, and, among them, some of the relevant ones are the conductivity and viscosity, according to several authors [41,45,46]. Therefore, if the addition of a compound, or a combination of them, is significantly affecting the final morphology of fibers, it might be as a consequence of modifying such solution parameters. Given that, as reported in studies, within moderate drug-loading ranges, fiber diameters generally increase with decreasing conductivity or increasing viscosity values [40,41,45,47,48], the combination of compounds that was used in the present work may be affecting the electrospinning solution either way, but most probably by increasing the viscosity of the solution, since none of the molecules employed was either a salt or a polyelectrolyte [40,41].

### 3.2. Determination of the Encapsulated Content and Its Stability over Time

To quantify the amount of encapsulated therapeutic agent, they were separated by gas chromatography, and then identified with mass spectrometry. In the calibration process, the elution times were precisely determined for the four compounds: methyl salicylate, 12.5 min; salicylic acid, 14.0 min; capsaicin, 29.0 min; and dihydrocapsaicin, 29.3 min (for further details in this regard, see Figure S1 in the supplementary information).

**Figure 2:**
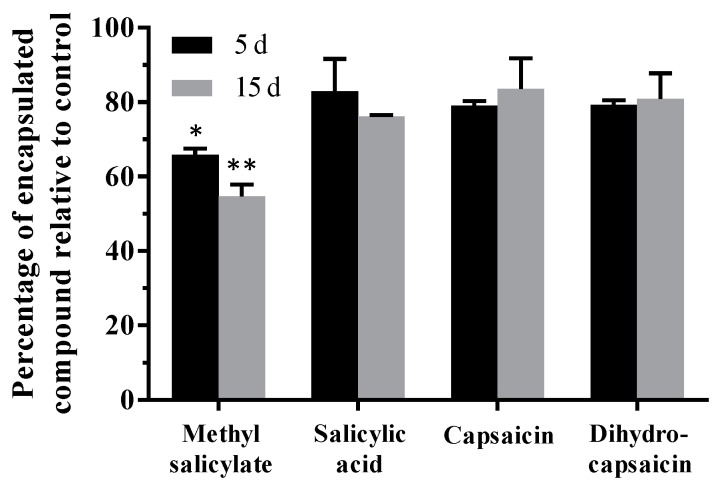
Stability of the loaded compounds over time. The graph indicates the percentage of compound that remained encapsulated in the nanofibers 5 and 15 days (d) after they were produced. Compound content was determined by GC-MS. Values are relative to the content measured right after the production of the nanofibers. Statistical analysis comprised multiple *t* test corrected for multiple comparisons by using the Holm–Sidak method. Significant changes in the content of each compound over time with respect to the corresponding initial values are indicated as: *, *p* < 0.05 and **, *p* < 0.01.

By following this procedure, the encapsulation efficiency was calculated for each therapeutic agent, which was found to be approximately 100% (in comparison to theoretical full encapsulation) in all cases when measuring right after the production of nanofibers (*t* = 0, data not shown). Such value was used as a reference for calculating the encapsulated content over time (Figure 2). Regarding the permanence of the encapsulated content, the obtained results indicate that methyl salicylate is lost over time; specifically, after 15 days, just 60.2 ± 7.8% of methyl salicylate remained in fibers. Regarding the other agents, there seemed to be a tendency to decrease the quantity of encapsulated compound, but these changes were not significant in the period of time that was analyzed (0–15 days).

Thus, by adjusting the operational parameters, it is possible to load the nanofibers described here with approximately 100% efficiency by using a concentration of 3.5% *w*/*w* of the three compounds, all together. In addition, the absence of significant changes in size and shape suggests that their encapsulation capacity might be higher. In this sense, by using the same nanostructured system, they were loaded with up to 16.6% *w*/*w* of 5-aminolevulinic acid in PMVE/MA-ES and Ac nanofibers [26]. Other evidence of electrospun nanofibers made of a blend of other polymers and PMVE/MA were shown to be able to be loaded with even zinc oxide-containing nanoparticles [33] and silver nanoparticles [34]. Indeed, the electrospinning technique allows loadability concentrations of up to 60% *w*/*w* in some cases [49] and encapsulation yields close to full efficiency levels [26,50].

Regarding the concentration of each therapeutic agent proposed, salicylic acid is usually used at concentrations of 0.5–60% *w*/*w* for the treatment of psoriasis [51]. The concentration that was chosen in this work was 1.5% *w*/*w*, which is similar to the concentrations used in acne creams [52]. The use of methyl salicylate in nanostructures has not been described yet, but it is in other topical applications to treat pain in general, especially musculoskeletal pain [21]. The concentrations of this compound used in creams are usually 10–30% *w*/*w* (*Bengay* and *Icy Hot* creams) and even 45% in the case of the *Reflex* gel. In the case of capsaicin, it has been used at 1% *w*/*w*, as in the present study, with respect to the base polymer in nanofibers and nanoparticles for topical use in psoriasis [53,54]. In addition, there is a commercial patch of 8% capsaicin, *Qutenza Astellas*, which is used to treat neuropathic pain.

One of the most surprising results was the loss of product observed for methyl salicylate, which seems to be due to the increase in surface area with respect to the volume that occurs in one-dimensional materials when they are nanostructured, which increases the evaporation of the encapsulated substances [55]. This effect would also be increased in those more volatile molecules, such as in the case of methyl salicylate, which is liquid at room temperature (melting point (mp): −9 °C), while capsaicin and salicylic acid are solid (mp: 62 and 159 °C, respectively). In any case, this loss of compound could be avoided or delayed by packaging the nanofiber mats tightly and hermetically, as is done for other similar products with volatile compounds. On the contrary, this might be an advantage for topical applications because of the possibility of gradually alternating the release of different compounds. From the *in vitro* skin permeability studies performed in our previous work to evaluate the potential of this polymeric family for topical applications [26], it was concluded that nanofibers made with this same material can achieve both fast and high rates of transdermal release of a non-volatile compound (90% of 5-ALA release in two hours by following a Higuchi kinetic model). However, under these conditions that mimic those existing in the human dermal surface (high humidity and 32.5 °C), the diffusion of a more volatile compound, such as methyl salicylate, should be faster, anticipating its treatment with respect to the less volatile ones.

### 3.3. Assessment of the Activation Capacity of TRPV1 by the Encapsulated Agents

The activity of the TRPV1 agonist compounds encapsulated in the experimental nanofibers (only methyl salicylate and capsaicin, not salicylic acid), alone or in combination with the other two agents (i.e., all three compounds), was assessed by treating HEK293-VR1 cells. These results were represented in percentages relative to the highest value obtained for the activation induced by capsaicin at 10 μM and using the lowest one after inoculating RR as the baseline.

**Figure 3:**
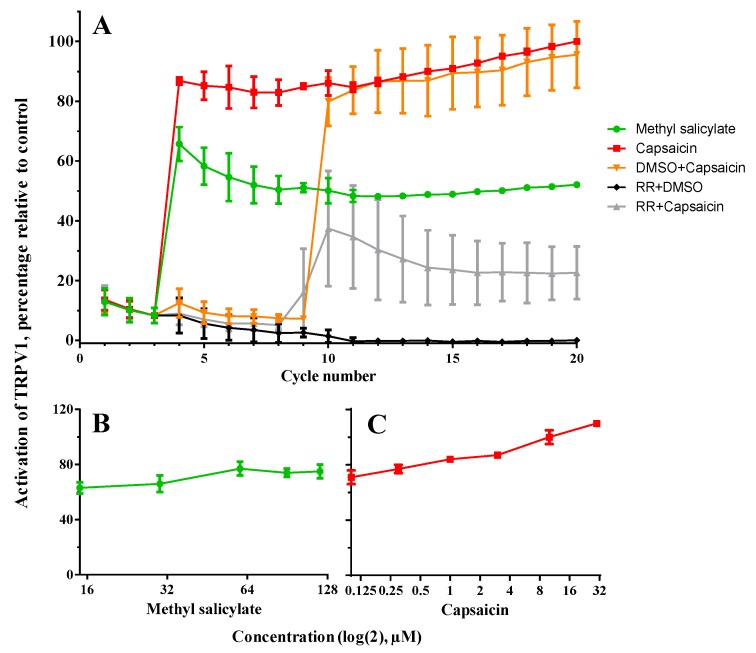
Functional analysis of transient receptor potential cation channel 1 (TRPV1) *in vitro* and calibration of its agonists. (**A**) TRPV1 activation dynamics were determined by performing fluorescence time-course assays in cells stably expressing TRPV1 after treatment with agonists (30 μM of methyl salicylate and 10 μM of capsaicin) and antagonist (ruthenium red, RR). TRPV1 activation was observed once agonists were added, while the inoculation of the antagonist attenuated this response. (**B**,**C**) Additionally, concentrations of agonists lower and higher than those to be tested from produced nanofibers under these experimental conditions were assayed in order to better assess the functionality of the compounds when they were encapsulated. For all analysis, the level of activation is calculated as the percentage relative to the highest value from the time-course in cells treated with 10 μM of capsaicin, and relative to the lowest value from RR treatment as the baseline.

Firstly, in Figure 3A, records of values induced by control samples to check the validity of the system are shown. As can be observed, both methyl salicylate and capsaicin are able to activate TRPV1 by inducing an increase of fluorescence because of the internal increase of Ca^2+^ after being inoculated in cycle three. The graph also shows that TRPV1 is highly activated right after the inoculation of each agent and remains at high levels, which even increase in the case of capsaicin. The specificity of the system is checked by the blocking of the channel with RR, which is even able to desensitize it to a subsequent stimulation with capsaicin (cycle 10). Figure 3 also shows that the activation of TRPV1 is concentration-dependent for both compounds (Figure 3B and 3C for methyl salicylate and capsaicin, respectively), considering the highest value from each time-course analysis for the calculations. In the specific case of methyl salicylate, this curve seems to plateau within the range of concentrations used, from 60 μM in particular.

**Figure 4:**
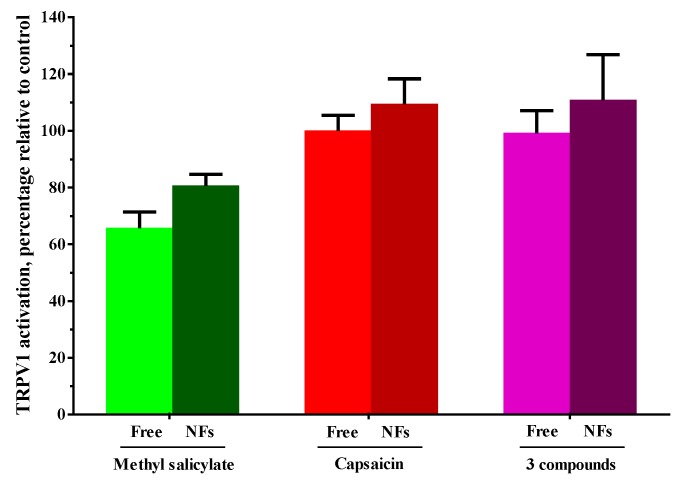
Effect of the encapsulation on the ability of the experimental compounds to activate TRPV1. Fluorescence time-course assays were performed on cells stably expressing TRPV1 and treated with the agonist compounds employed either alone or in combination, and also including salicylic acid (three compounds). These samples included compound-containing nanofibers (NFs) and non-encapsulated compounds (Free). The level of activation is calculated as the percentage relative to the highest value from the time-course in cells treated with 10 μM of capsaicin and the lowest value from RR treatment. Statistical analysis comprised two-way ANOVA corrected with Tukey’s test for multiple comparisons (Free vs. NFs) and no significant differences were found between the experimental and control samples.

For the comparison of the activity of the agonist compounds on TRPV1 when they are encapsulated in nanofibers, alone or together with the other therapeutic agents, the same above-described procedure was followed, but including experimental nanofibers that were previously dissolved in DMSO. In these *in vitro* cell assays (Figure 4), although the obtained values were moderately higher when the agent was encapsulated, no significant changes were found between the encapsulated and non-encapsulated compounds, and only significant variations were found, as expected, when comparing each compound among them (two-way ANOVA corrected with Tukey’s test for multiple comparisons; F (3, 8) = 15.59; *p* = 0.0011), meaning that the encapsulation process did not affect the chemical properties of the encapsulated agents. Some activation of TRPV1 was also observed when treating with empty nanofibers.

The combination of different compounds within the same formulation generally pursues a synergistic effect that could allow the reduction of the concentration of each one, and thus their potential adverse effects; however, other benefits from such an approach might be the combination of other functional properties such as, in this case, the ones introduced previously, which are also related to treating psoriasis and other factors such as their antimicrobial activities [56,57].

**Figure 5:**
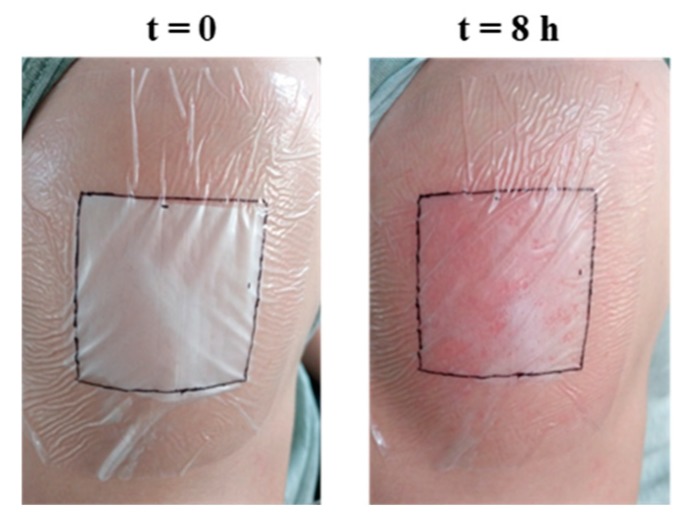
Preliminary assessment of the feasibility of transdermal patches based on experimental nanofibers. Dressings for topical applications produced by electrospinning experimental nanofibers onto adhesive patches were developed and tested for up to eight hours on volunteers’ arms. Representative pictures of their application are shown at both the beginning and the end of the test. After eight hours of treatment, the degradation of the nanofibers and the typical reddening of the skin as a consequence of the action of capsaicin on the surface of the skin were observed.

Finally, but also related to the assessment of the ability of the developed nanofibers to activate TRPV1, it has been possible to successfully electrospin the experimental nanofibers on adhesive dressings for topical application and preliminarily check their gradual degradation by visual observation, as well as their content release by assessing the typical reddening reaction of the capsaicin in the skin (Figure 5). Both effects were evident eight hours after the application of the patch covered with loaded nanofibers on the skin from volunteers’ arms. In this sense, no effects were either observed or detected on skin when treating with control patches based on empty nanofibers. Future studies will be aimed at describing the release and dermal absorption of this system, as well as at revealing the possible effect of the polymers on TRPV1.

## 4. Conclusions

In this work, it has been possible to optimize the electrospinning parameters to create PMVE/MA-ES nanofibers loaded with a combination of three compounds with therapeutic activity to alleviate the dermal symptoms of psoriasis (salicylic acid, methyl salicylate, and capsaicin at a concentration of 3.5% *w*/*w* all together with respect to the amount of polymer), with an encapsulation efficiency of approximately 100%. The stability studies of the content encapsulated over time (up to 15 days after its production) showed significant losses especially of methyl salicylate, which were probably due to its higher volatility. *In vitro* studies revealed that the TRPV1 agonist compounds maintained their channel-activating capacity at the expected levels after being encapsulated. Finally, it was found that the proposed nanofibers are suitable for the creation of skin adhesive dressings and allow the release of the encapsulated compounds by the disintegration of the nanostructure. In this study, the effect of those compounds with the capacity to activate TRPV1 was checked *in vivo*.

## Supplementary Materials

The following are available online at http://www.mdpi.com/1999-4923/11/1/14/s1, Figure S1: GC-MS chromatograms and calibration curves.
