# Supplementary material for: Development of A New Delivery System Based on Drug-Loadable Electrospun Nanofibers for Psoriasis Treatment
Source: Pharmaceutics. 2019 Jan 4;11(1):14. doi: 10.3390/pharmaceutics11010014 (PMC6359116; doi:10.3390/pharmaceutics11010014)
Supplement: Supplementary file 1 [file pharmaceutics-11-00014-s001.pdf]

pharmaceutics

Supplementary Materials: Development of A New Delivery System Based on Drug-Loadable Electrospun Nanofibers for Psoriasis Treatment

Leticia Martínez-Ortega, Amalia Mira, Asia Fernandez-Carvajal, C. Reyes Mateo, Ricardo Mallavia and Alberto Falco

1

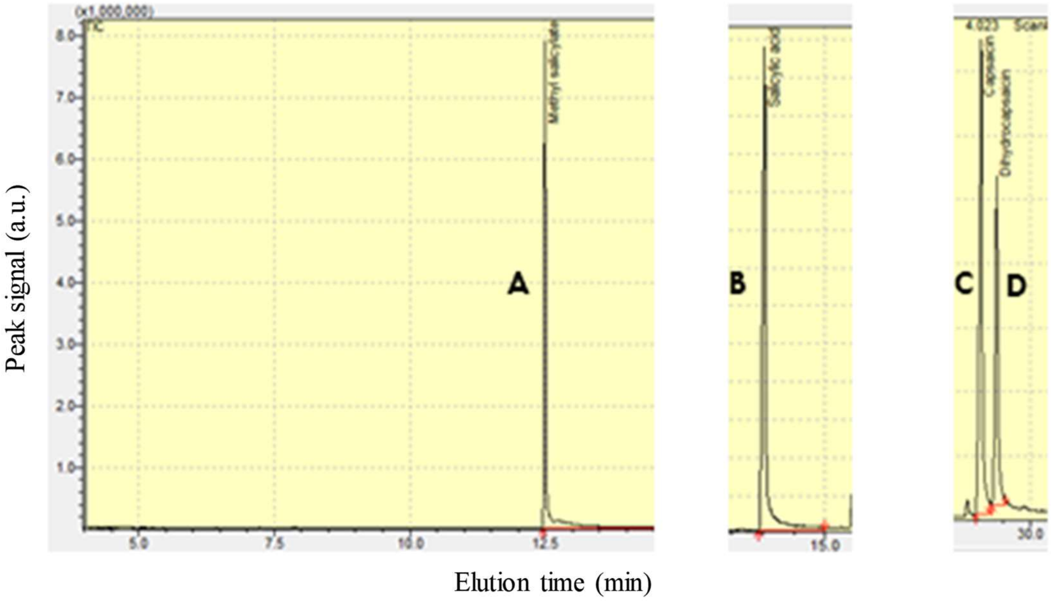

2

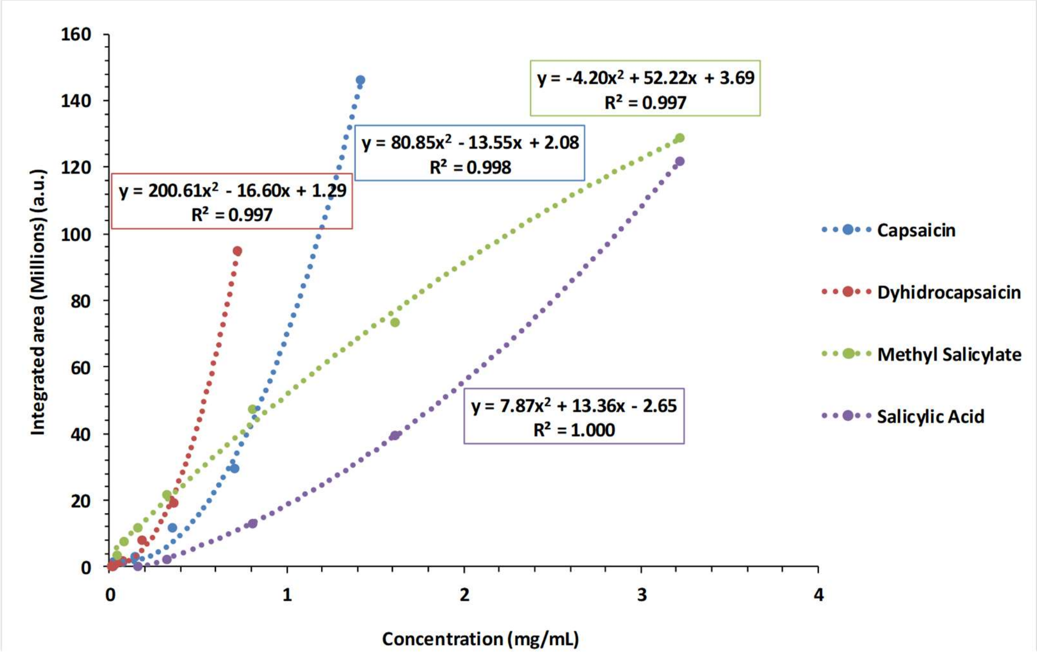

Figure S1. GC-MS chromatograms and calibration curves. By following the described methodology, chromatograms were obtained similar to the one shown in collage image 1, which comprises each compound peak at its corresponding elution time (A, methyl salicylate; B, salicylic acid; C, capsaicin; D, dihydrocapsaicin). Since the area shaping each peak correlated to the amount of compound used, it was possible to create calibration curves such as the representative ones shown in the graph (2), which preferably adjusted to quadratic equations with R^2 coefficients greater than 0.99 in all cases.
